# Personal value, self-efficacy, and social acceptability of a social behavior as correlates of behavioral action in social anxiety

**DOI:** 10.47626/2237-6089-2020-0129

**Published:** 2021-10-22

**Authors:** Carol S. Lee, Christina Yeghiazarian

**Affiliations:** 1 Nevada State College Henderson NV USA Nevada State College, Henderson, NV, USA.

**Keywords:** Social anxiety, behavioral action, self-efficacy, values, exposure

## Abstract

**Introduction:**

Current therapies for social anxiety disorder emphasize taking behavioral action; active engagement of a behavior despite any present fear or anxiety that is associated with the behavior, through use of exposures. However, less is known about the mechanisms of behavioral action. The present study aimed to examine personal value, self-efficacy, and the perceived social acceptability of a social behavior as correlates of behavioral action in a high social anxiety sample.

**Method:**

The present study utilized vignettes and self-report measures to examine self-efficacy, personal value, and the social acceptability of a social behavior as correlates of behavioral action in a high social anxiety sample (N = 92).

**Results:**

The findings indicated that self-efficacy, but not personal value or social acceptability, was significantly associated with social anxiety. Additionally, with all variables included in the multiple regression model, social anxiety was significantly associated with behavioral action, while personal value and self-efficacy were associated with behavioral action over and above social anxiety.

**Discussion:**

The results highlight the potential for self-efficacy and personal value as target mechanisms for increasing engagement with exposures and behavioral experiments in treatments for social anxiety.

## Introduction

According to models of social anxiety disorder (SAD), one of the key maintenance factors of SAD is behavioral avoidance.^1-3^ Individuals with SAD typically avoid social situations, as simply removing the feared stimulus eliminates the accompanying fear as well. However, while avoidance initially decreases anxiety, it later serves to maintain or exacerbate symptoms. To target this phenomenon, current therapies for SAD emphasize taking behavioral action,^4^ which is active engagement of a behavior despite any present fear or anxiety that is associated with that behavior.^5,6^ In effect, the literature consistently highlights actively engaging with a behavior as an important mechanism of a greater cycle of change in psychopathology. However, little attention has been paid to the specific predictors of behavioral action in the context of social anxiety.

One way to examine this may be to consult current social psychology models of behavior, which present specific predictors of a given behavior.^7-9^ One such model of behavior is the Theory of Planned Behavior (TPB),^7,10,11^ which social psychologists have identified as being arguably the most prominent, widely applied, and widely cited model of intentional behavior to date.^12^ According to the TPB, behaviors are most likely to occur: if the behavior or its outcome is personally valued, if the behavior is perceived to be socially acceptable, and if individuals have greater self-efficacy for the behavior. Here, self-efficacy is defined as belief in one’s ability to perform the behavior successfully. Although the TPB does not integrate emotional contexts or psychopathology into the model, it has been applied to examine the engagement of several mental health related behaviors. Notably, such studies consistently indicate that, of all the predictors in the TPB, the strongest predictors of the behaviors examined were the personal value of behaviors and self-efficacy.^13-18^

While the TPB has not been examined in the context of behavioral action in social anxiety, there is evidence that beliefs about self-efficacy, personal value, and the social acceptability of the behavior are relevant to our understanding of behavioral action in social anxiety. For example, with regard to self-efficacy, studies indicate that there is a negative association between greater social self-efficacy and social anxiety.^4,19-22^ Additionally, self-efficacy has been shown to be negatively associated with social avoidance,^22^ and positively associated with behavioral action over and above social anxiety.^4^ Further, studies have indicated that changes in social self-efficacy predict changes in social avoidance,^19,20^ and that self-efficacy predicts engagement in a speech task.^22^

With regard to personal values, studies indicate that therapeutic practices such as acceptance-based behavior therapies (ABBTs), a primary component of which is clarifying and acting in accordance to one’s values, predict decreased social anxiety and an increased likelihood of the occurrence of social anxiety-provoking behaviors.^23-29^ However, these studies do not explicitly examine the relations between personal value, social anxiety, and behavioral action. Interestingly, the only study to date that did explicitly examine these relations found that while personal value did predict behavioral action, it was not associated with social anxiety, suggesting that personal value has an effect on behavioral action independent of social anxiety.^29^ Taken together, the findings described here may suggest that although ABBTs are an effective treatment for SAD, the personal values component targets individuals’ avoidance and action, rather than their anxiety.

Finally, with regard to the social acceptability of a behavior, a significant body of literature indicates that the perceived social acceptability of a social behavior is positively associated with that behavior.^30-33^ Additionally, studies and theoretical models indicate a positive association between social anxiety and the social acceptability of a social behavior (e.g. blushing while giving a speech).^2,34,35^ Here, the positive associations between social anxiety and social acceptability and the associations between social acceptability and social behavior may indicate that perceived social acceptability is positively associated with the likelihood of a social anxiety-provoking behavior (behavioral action).

In sum, while the literature indicates that self-efficacy for a behavior is negatively associated with social anxiety, there is little empirical evidence on the relations between social anxiety and personal value or the perceived social acceptability of a behavior. Further, these factors have not been examined in a single model. Additionally, while the literature indicates that self-efficacy, personal value, and perceived social acceptability each predict social behaviors, there is little research on this effect in the context of social anxiety and no research examining each of these constructs in the same model. To address these issues, the present study investigated self-efficacy, personal value, and perceived social acceptability of a behavior as correlates of both social anxiety and behavioral action in the context of social anxiety. We examined this cross-sectionally, using a series of short vignettes about social situations. Additionally, given the literature indicating that depression can increase social withdrawal and isolation, resulting in decreased social behavior despite social anxiety, we also chose to control for symptoms of depression.^36,37^ We hypothesized that (1) when examined together, greater perceived social acceptability and self-efficacy, but not personal value, would each be uniquely associated with lower levels of social anxiety and that (2) lower levels of social anxiety would be associated with behavioral action, while greater personal value, the perceived social acceptability of the behavior, and self-efficacy would be associated with a greater likelihood of behavioral action over and above social anxiety.

## Method

### Participants

This study was approved by the institutional review board (IRB). The online Qualtrics survey was advertised on an open-access four-year college campus using flyers and electronic messages advertising a study of “social situations.” Although we only advertised on the college campus, the study was open to all individuals meeting the inclusion criteria. The inclusion criteria were as follows: individuals aged from 18 to 65 years and pre-screened for high social anxiety. Individuals were considered to have high social anxiety if they had a Social Phobia Inventory (SPIN) score equal to or greater than the established and recommended clinical cut-off score of 19.^38^ If less than 80% of the items on 80% of the measures were answered, the participant’s data were excluded; thus, 2 individuals were dropped from the analysis. There were no other missing data. To control for random responses, if participants answered a manipulation check question incorrectly, we dropped the preceding vignette as missing data. As a result, 8 individuals were dropped from our total sample. We recruited a total of 102 participants; the final sample consisted of 92 participants, the demographic characteristics of whom can be seen in Table 1. In exchange for their participation, participants were offered extra credit in participating classes.

**Table 1:** Participant demographics

Demographic	Result
SPIN, mean (SD)	34.36 (10.16)
Age, mean (SD)	25.49 (8.04)
Gender, n (%)	
Male	14 (15.22)
Female	75 (81.52)
Other*	3 (3.26)
Sexual orientation, n (%)	
Gay/lesbian	5 (5.43)
Bisexual	14 (15.22)
Heterosexual	68 (73.91)
Other^†^	4 (4.35)
Racial identity, n (%)	
Alaskan native	0 (0)
Asian	7 (7.61)
Black	8 (8.70)
Latinx	35 (38.04)
White	28 (30.43)
Multiracial/other^‡^	14 (15.22)

SD = standard deviation; SPIN = Social Phobia Inventory.

* Other identities = agender, non-binary, prefer not to respond.

^†^ Other identities endorsed = queer, questioning, prefer not to respond, unknown.

^‡^ Other identities = prefer not to respond.

### Assessment measures

#### Depression, Anxiety, and Stress Scales – Depression Subscale (DASS-D)36

The DASS-D is a 7-item self-report measure evaluating symptoms of depression. Respondents rated how much each item applied to them, on a scale from 0 (did not apply to me at all) to 3 (applied to me very much or most of the time). We utilized DASS-D to control for participants’ depression, as depression can influence social engagement and perception.^36^ The DASS-D has been shown to have good convergent and discriminant validity and good internal consistency (α = 0.72).^39^ Similarly, the DASS-D in our sample displayed good internal consistency (α = 0.86).

#### Social Phobia Inventory (SPIN)38

The SPIN is a self-report measure consisting of 17 statements that evaluate fear, avoidance, and physiological discomfort around social situations (sample items: “Parties and social events scare me”; “I avoid having to give speeches”; “Trembling or shaking in front of others is distressing to me”). Participants are asked to rate the relevance of each statement over the past week on a 0 to 4 point Likert-type scale (Not at all, A Little Bit, Somewhat, Very Much, Extremely). Higher scores indicate higher social anxiety. According to the literature, based on a receiver operating characteristic (ROC) analysis examining the clinical sensitivity and specificity of proposed cut-off scores, individuals with scores of 19 or higher are likely to experience social anxiety at the disorder level. The SPIN was administered to assess participants’ social anxiety. The SPIN has been shown to have good test-retest reliability, excellent internal consistency (α = 0.94), and good convergent and discriminant validity.^38^ Similarly, the SPIN in our sample displayed good internal consistency (α = 0.81).

#### Additional questions

As a part of the demographic questionnaire, individuals were asked to indicate if they had ever been diagnosed with an Asperger’s or autism spectrum disorder (ASD), as symptoms of these disorders can influence social engagement or perceptions.^40^ No participants endorsed a history of ASD.

## Vignettes

We presented participants with a series of eight vignettes, taken from a similar vignette study conducted by Lee and Hayes-Skelton.^4^ These vignettes were specifically designed to reflect either a social interaction (going to a party, talking with a classmate you do not know very well, talking to people you know and do not know at a party, and disagreeing with a classmate) or a social performance situation (giving a speech, answering a question in class, giving a brief report, and getting on stage to receive an award) and determined to be situations with the highest fear and avoidance ratings by individuals with SAD in an archival dataset. For each vignette, participants were asked to answer a series of four questions using a 0 to 8 Likert-type scale adapted from Lee and Hayes-Skelton.^4^ The first question assessed participants’ personal value of taking the behavioral action (“In this situation, how important is it to you that you…”), the second question assessed participants’ perceived social acceptability of the behavioral action (“In this situation, how “normal” or socially acceptable would it be for you to…”), and the third question assessed participants’ self-efficacy to take the behavioral action successfully (“One reason some people have difficulties in certain social situations is because they do not believe they have the skills necessary to do a good job. In this situation, how confident are you that you are able to ___ successfully?”). The final question asked participants to rate their likelihood of taking the behavioral action identified in the vignette (“How likely are you to…”).

## Vignette scoring

We totaled participants’ answers to all eight vignettes to yield separate total scores for personal value, social acceptability, self-efficacy, and behavioral action. The total scores yielded acceptable to good internal consistencies (α = 0.70, 0.85, 0.71, and 0.70, respectively).

## Manipulation check

After each vignette, we asked participants a manipulation check question, asking “what was the situation about?” Participants who answered incorrectly had that vignette’s answers dropped from the analysis. Participants who failed at least one manipulation check were dropped from the study, leading to eight participants being dropped from the final analysis.

## Procedures

Participants provided informed consent for this IRB approved study, before completing a comprehensive demographics questionnaire, followed by the vignette survey, the SPIN, and the DASS.

## Results

### Preliminary analyses

The means, standard deviations, and correlation matrix for all measures are presented in Table 2. All variables were examined for assumptions of normality. All skewness and kurtosis values were within acceptable ranges.^41^ In addition, we examined personal value, social acceptability, self-efficacy, SPIN, and DASS-D by age, race/ethnicity, and gender using a series of ANOVAs and found no significant differences between groups in any of these variables (ps = 0.07 - 0.80).

**Table 2:** Means, standard deviations, and correlations among study variables

	Mean (SD)	SPIN	DASS-D	Personal value	Social acceptability	Self-efficacy	Behavioral action
SPIN	34.37 (10.16)	1.00	0.19	-0.08	-0.05	-0.27*	-0.39^†^
DASS-D	7.88 (5.11)	-	1.00	-0.04	-0.08	-0.04	-0.12
Personal value	29.85 (9.40)	-	-	1.00	0.39^†^	0.29^†^	0.55^†^
Social acceptability	37.79 (11.97)	-	-	-	1.00	0.62^†^	0.51^†^
Self-efficacy	34.89 (9.97)	-	-	-	-	1.00	0.76^†^
Behavioral action	34.35 (10.03)	-	-	-	-	-	1.00

DASS-D = Depression, Anxiety, and Stress Scales – Depression Subscale; SD = standard deviation; SPIN = Social Phobia Inventory.

* p < 0.05; ^†^ p < 0.01.

### Hypothesis 1

To test the hypothesis that social acceptability and self-efficacy, but not personal value, would be negatively associated with social anxiety, we conducted a multiple regression analysis. Here, the independent variables were personal value, social acceptability, and self-efficacy, with DASS-D as a covariate, and the dependent variable was the SPIN (See Table 3). With all variables entered, the full model was significant and accounted for 14% of the variance of the SPIN (R^2^ = 0.14, F(4, 88) = 3.50, p = 0.01). In this model, self-efficacy was significantly associated with the SPIN (β = -0.36, p < 0.01), while personal value and social acceptability were not (β = -0.06, p < 0.01; β = 0.59, p = 0.08, respectively).

**Table 3:** Personal value, social acceptability, and self-efficacy as correlates of social anxiety (N = 93)

Variable	B	β	SE
DASS-D	0.41*	0.20	0.20
Personal value	-0.07	-0.06	0.12
Social acceptability	-0.20	0.23	0.11
Self-efficacy	-0.39^†^	-0.38	0.13
R^2^	0.14		

DASS-D = Depression, Anxiety, and Stress Scales – Depression Subscale; SE = standard error.

* p < 0.05; ^†^ p < 0.01

### Hypothesis 2

To examine personal value, social acceptability, and self-efficacy as correlates of behavioral action over and above social anxiety in the same model, we conducted a two-stage hierarchical regression. Here, the independent variables were personal value, social acceptability, self-efficacy, and the SPIN, with DASS-D as a covariate, and the dependent variable was behavioral action (See Table 4). In Step 1, we entered the SPIN and the DASS-D into the model. In Step 2, we entered personal value, social acceptability, and self-efficacy into the model. The hierarchical multiple regression revealed that the model was significant at Step 1, accounting for 15% of the variance (R^2^ = 0.15, F (2, 90) = 7.88, p < 0.001). Here, the SPIN was significantly associated with behavioral action, although the DASS-D was not (β = -0.38, p < 0.001; β = -0.04, p = 0.68, respectively). Adding personal value, social acceptability, and self-efficacy into the model at Step 2 contributed significantly more to the model, explaining an additional 58% of the variance in behavioral action (ΔR^2^ = 0.58, ΔF(5, 87) = 62.85, p < 0.001). The complete model, with all independent variables in the model, was significant and accounted for 73% of the variance of behavioral action (R^2^ = 0.73, F(5, 87) = 47.36, p < 0.001). In this model, the SPIN, personal value, and self-efficacy were significantly associated with behavioral action (β = -0.18, p < 0.01; β = 0.37, p < 0.001; β = 0.62, p < 0.001, respectively). However, the DASS-D and social acceptability were not (β = -0.05, p = 0.43; β = -0.04, p = 0.64, respectively).

**Table 4:** Social anxiety, personal value, social acceptability, and self-efficacy as correlates of behavioral action (N = 93)

Variable	B	SE	β	R^**2**^	ΔR^**2**^
Step 1				0.15	0.15^†^
DASS-D	-0.09	0.20	0.20		
SPIN	-0.38^†^	0.10	-0.38		
Step 2				0.73	0.58^†^
DASS-D	-0.09	0.11	-0.05		
SPIN	-0.18*	0.06	-0.18		
Personal value	0.40^†^	0.07	0.37		
Social acceptability	-0.03	0.06	-0.04		
Self-efficacy	0.63^†^	0.08	0.62		

DASS-D = Depression, Anxiety, and Stress Scales – Depression Subscale; SPIN = Social Phobia Inventory; SE = standard error.

* p < 0.01; ^†^ p < 0.001.

## Discussion

Although models of SAD implicate behavioral action as a key mechanism of change in psychopathology, there has been little attention to the factors associated with behavioral action itself. As noted above, one framework to better understand the mechanisms of behavioral action may be the TPB, which highlights self-efficacy, personal value, and the social acceptability of the behavior as predictors of general social behavior. However, these predictors have yet to be fully examined together in the context of behavioral action in social anxiety. As such, the present study investigated self-efficacy, personal value, and the perceived social acceptability of a behavior as correlates of both social anxiety and behavioral action in the context of social anxiety.

Results partially supported Hypothesis 1, which proposed that when examined together, greater perceived social acceptability and self-efficacy, but not personal value, would each be uniquely associated with lower levels of social anxiety. As predicted, self-efficacy was significantly associated with social anxiety. This is consistent with both models of SAD and studies indicating that self-efficacy is negatively associated with social anxiety.^1-4,19-22^ Our results also indicated that personal value was not associated with social anxiety. The findings here support Lee’s findings that when personal value is examined outside of the context of ABBTs, it exists independent from social anxiety.^29^ As noted by Lee,^29^ this explanation is consistent with acceptance-based theories that indicate that anxiety does not change a value, but rather gets in the way of individuals acting in accordance with their values.^42-44^

Contrary to our hypothesis, the perceived social acceptability of a behavior was not significantly associated with social anxiety. Although this appears to be in direct contrast to the existing literature in the field, the difference may be a result of the way our social situations were presented. For example, Dijk et al.^34^ presented social behaviors that were paired with a relatively ambiguous, yet typically feared performance outcome in individuals with SAD (e.g. blushing while giving a speech). In contrast, our behavioral actions were not paired with a feared performance outcome (e.g. giving a speech). When taken with models of SAD^1-3^ that indicate that individuals with SAD have more rigid beliefs about their performance in social situations, the results may indicate that while individuals with SAD find social behaviors to be socially acceptable, any perceived mistakes in social performance or the occurrence of feared outcomes are not.

Results partially supported Hypothesis 2, which predicted that lower levels of social anxiety would be associated with behavioral action, while greater personal value, and the perceived social acceptability of the behavior, and self-efficacy would be associated with a greater likelihood of behavioral action over and above social anxiety. As predicted, lower levels of social anxiety were significantly associated with behavioral action – a finding consistent with models of SAD^1-3^ and the empirical studies the models are built upon. Additionally, as hypothesized, both self-efficacy and personal value were significantly associated with behavioral action over and above social anxiety. This is consistent with the TPB,^11^ as well as with previous research indicating that, when individually examined, both are significantly associated with anxiety-provoking behavioral actions.^4,29^ In contrast, the perceived social acceptability of a behavior was not associated with behavioral action. Again, the unexpected findings here may be a result of our behavioral actions not being paired with a feared performance outcome. For example, it may be that the social acceptability of giving a speech does not impact the likelihood of giving the speech, but that the social acceptability of blushing while giving a speech does. As such, further research is necessary to examine the nuances of normative beliefs in the context of social anxiety before making further conclusions about its role in behavioral action.

Overall, the results indicated that greater personal value and greater self-efficacy, but not perceived social acceptability, were significantly associated with behavioral action over and above social anxiety. While the previous literature has examined the strength of these associations with behavioral action individually, they had not previously been examined together in the same model. Of note, the results indicated that self-efficacy was the strongest correlate of behavioral action, indicating that targeting self-efficacy may be the most effective way to target social anxiety-provoking behavioral actions. However, given self-efficacy’s strong association with social anxiety, such interventions may be limited in individuals extremely high in social anxiety. In contrast, personal value was not associated with social anxiety, supporting previous findings that it may impact behavioral action independently from social anxiety. As such, personal value may be an effective way to increase social anxiety-provoking behavioral actions regardless of social anxiety severity. To examine this, further research should examine both self-efficacy and personal value experimentally as predictors of behavioral action in individuals high on social anxiety.

### Limitations and future directions

Our results should be considered in the context of several limitations. First, the study is cross-sectional, rather than experimental. As such, our data does not establish temporal precedence between measures and cannot yield causal conclusions. Although the proposed directionality of our model fits with current theoretical models of behavior and social anxiety,^2,11^ temporal precedence is necessary to make inferences about directionality. Second, our study used vignettes, which cannot replace real-world social situations, thereby reducing external validity. Specifically, one concern is that participants may have rated their likelihood to take the behavioral action based on how they believed they should react, rather than how they would react. As such, it is possible that participants overestimated their likelihood of taking the behavioral action. However, given the moderate mean (mean = 34.35, SE = 10.03) and the wide range (4-58) of responses, it is unlikely that there is this ceiling effect in behavioral action. Future studies could address this limitation examining behavioral action in vivo. Additionally, our measures of self-efficacy, personal value, and social acceptability were not assessed for reliability or validity, outside of internal consistencies and ratings of face validity. To address this limitation, future studies should more formally evaluate the reliability and validity of measures.

Finally, our sample consisted of predominantly Latinx or White, heterosexual, cisgender women. Given the literature indicating that members of nondominant groups experience additional anxiety, particularly when interacting with members of the dominant space,^45,46^ as well as the literature on the cultural differences in social anxiety,^47,48^ our results cannot be adequately generalized to other identities. To do so, future research should include a more diverse sample.

### Clinical implications

Despite its limitations, the current study adds to the present literature on behavioral treatments for SAD by highlighting potential mechanisms leading to engagement with feared stimuli. For example, exposures, which involve individuals facing or being exposed to feared social stimuli, emphasize increasing behavioral action and decreasing behavioral avoidance. As such, our results may suggest that increasing self-efficacy is the most effective way to increase engagement in exposures through known methods of increasing self-efficacy such as modeling, encouragement, and practice.^49^ However, because one’s ability to increase self-efficacy is likely impacted by social anxiety severity, doing so may prove challenging in clients with higher levels of SAD. Encouragingly, since personal value is not associated with social anxiety, another method of increasing engagement in exposures may be to increase personal value regardless of social anxiety severity. For example, clinicians might consider utilizing values work to explicitly frame exposure engagement as a valued action.
